# Unfinished Tasks and Unsettled Minds: A Diary Study on Personal Smartphone Interruptions, Frustration, and Rumination

**DOI:** 10.3390/bs15070871

**Published:** 2025-06-26

**Authors:** Daantje Derks, Heleen van Mierlo, Clara Kühner

**Affiliations:** 1 Department of Work and Organizational Psychology, Erasmus University Rotterdam, 3062 PA Rotterdam, The Netherlands; vanmierlo@essb.eur.nl; 2 Wilhelm Wundt Institute for Psychology, Leipzig University, 04109 Leipzig, Germany; clara.kuehner@uni-leipzig.de

**Keywords:** smartphone interruptions, personal smartphone use, unfinished tasks, work-related rumination, boundary-crossing behavior

## Abstract

Personal smartphone use at work stretches the boundary between professional and personal life, leading to a more fragmented workday. This study investigates how interruptions from personal smartphone use during work hours affect employees’ performance and well-being. Our primary aim is to clarify the pathways through which personal smartphone interruptions impact employee well-being, as reflected in work-related rumination after work (affective rumination, problem-solving pondering, and psychological detachment). Integrating propositions of social role theory and action regulation theory, we hypothesize that such interruptions undermine task accomplishment, which, in turn, increases feelings of frustration. Furthermore, we propose that frustration explains the link between reduced task accomplishment and increased work-related rumination after work. To test these hypotheses, we conducted a quantitative daily diary study with 91 employees from diverse occupations, collecting data over five consecutive workdays, including between 354 and 399 observations per day. Multi-level analyses revealed that interruptions from personal smartphone use indirectly increased frustration by undermining task accomplishment. Additionally, frustration fully mediated the relationship between task accomplishment and work-related rumination in the evening. These findings highlight the importance of managing personal smartphone use at work to protect employee performance and well-being. We conclude by critically examining the broader theoretical significance and practical applications of our findings.

## 1. Introduction

Smartphones have become the standard communication tool in both business and private communication. They enable us to constantly connect with work contacts as well as with family and friends (Messenger & Gschwind, 2016). In the Netherlands, 96% of the adult population possesses a smartphone, which they use for work, private purposes, or both. Approximately 60% admit using their smartphone for private purposes during worktime (Deloitte, 2017). The constant connectivity facilitated by smartphones provides important organizational benefits like increased autonomy and flexibility but also produces unintended side effects like triggering interruptions to the workflow by, for example, incoming phone calls or e-mail notifications (Addas & Pinsonneault, 2015). Interruptions facilitated by communication technology use are frequent and have an important impact on work since they undermine job performance (e.g., Andreassen et al., 2014; Leroy, 2009; Weatherbee, 2010).

Interruptions can have many forms; they can occur at work or in the private realm, and their content can be work-related or private. While work-related interruptions, both at work and in the private domain, have been subject to substantial research, much less is known about interruptions originating from the home domain during worktime (for exceptions, see Derks et al., 2021; Sonnentag et al., 2018). Due to the intensive use of mobile communication technologies (i.e., smartphones), most employees integrate their work and home domains to some extent, creating a work context in which boundary-crossing behavior is ubiquitous. This implies being available for work during off-job hours and being available for family and friends during work hours (Nilsen et al., 2024). A previous study indicated that this boundary-crossing behavior—operationalized as interruptions by personal smartphone use during worktime—is associated with certain costs, such as feeling interrupted and exhausted (Derks et al., 2021).

There is ample evidence that interruptions at work can undermine the workflow, create additional workload, time pressure, and strain (Lin et al., 2013), and negatively affect task accomplishment (e.g., Baethge & Rigotti, 2013; Bailey & Konstan, 2006; Hacker, 2005). The time spent on personal smartphone use during worktime automatically implies that time dedicated to fulfilling work tasks is reduced (Wan et al., 2019). However, as Patterer et al. (2021) already acknowledged, personal smartphone use also has a potential bright side in the social benefits it may provide as it can help employees to attend to their personal roles in life more easily. This can result in a feeling of control since it contributes to managing both your work and nonwork responsibilities simultaneously. Nevertheless, the costs of this were mainly attributed to the work domain as functioning at work was impeded, resulting in negative work–nonwork interference (Patterer et al., 2021). In addition, interruptions to the workflow are associated with feelings of irritation and frustration (Konradt et al., 2003; Leroy & Glomb, 2018). Building on this, in the current study, we integrate these perspectives on personal smartphone interruptions, proposing that it is by undermining the workflow and compromising task accomplishment that these interruptions contribute to feelings of frustration. In other words, being interrupted by personal smartphone use makes it harder to complete daily tasks as planned, leaving the employee feeling frustrated at the end of the workday.

Subsequently, a lack of task accomplishment and the ensuing frustration at the end of the workday may spill over to the home domain. There is some initial evidence that lack of task accomplishment (i.e., unfinished tasks) is a trigger for work-related rumination in the evening (Syrek et al., 2017; Weigelt et al., 2019b). And frustration at work seems to be related to both poor psychological detachment from work and negative work reflections in the evening (Cropley & Zijlstra, 2011). When employees are unable to switch off during their off-job time or engage in work-related rumination, this may have important consequences for recovery (Kinnunen et al., 2017), mental health (e.g., more exhaustion, poor sleep, and lower life satisfaction), and physical health (e.g., higher physical discomfort and more fatigue) in the long run (see Wendsche & Lohmann-Haislah, 2017, for a meta-analysis). Therefore, it is important to examine whether, next to diminished task accomplishment, frustration is a potential mechanism linking personal smartphone interruptions during the workday to work-related rumination after working hours.

### Aims and Contributions

This study investigates how personal smartphone interruptions at work spill over to the home domain, affecting employee well-being. We propose that personal smartphone interruptions at work leave people feeling frustrated at the end of the workday, as explained by reduced task accomplishment. In turn, we examine how task accomplishment and end-of-day frustration contribute to work-related rumination in the evening, distinguishing between affective rumination, problem-solving pondering, and psychological detachment. To capture the day-to-day variability of these processes, we conducted a multi-level experience sampling study across multiple workdays.

This study offers two main contributions. First, it extends the literature on technology-mediated interruptions (e.g., A. Chen & Karahanna, 2014) by focusing on perceived interruptions from personal smartphone use during work hours (Derks et al., 2021). We examine task accomplishment as a mechanism linking these interruptions to frustration, exploring how daily cumulative interruptions may indirectly increase frustration by undermining task completion.

**Figure 1:**
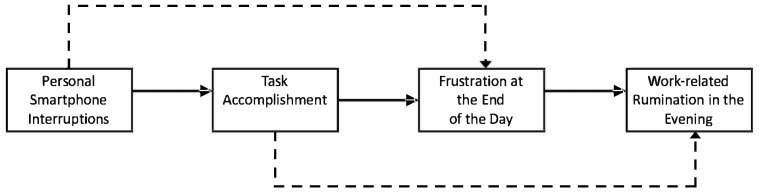
Research model.

Second, we advance the literature on work-related rumination in two ways: responding to Cropley and Zijlstra’s (2011) call to examine all three types of rumination—affective rumination, problem-solving pondering, and psychological detachment—and investigating end-of-day frustration as a mediator between task accomplishment and rumination. Figure 1 presents the conceptual model.

## 2. Theoretical Background

### 2.1. The Smartphone as a Tool to Manage Multiple Roles

Social role theory holds that people juggle several roles, each with its own norms, expectations, and behavior (Biddle, 1986). Although the workplace usually activates the work role, a message from a friend or family member can instantly shift employees to their private role, making personal smartphone use at work a cross-role interruption (Anderson et al., 2019; Kossek et al., 2012). Many employees feel compelled to answer such messages, even on the job (Carton & Aiello, 2009).

Smartphones make the work–home boundary more flexible and permeable (Kühner et al., 2023; Ramarajan & Reid, 2013; Reyt & Wiesenfeld, 2015). People differ in how much domain integration they prefer (Ashforth et al., 2000), and organizations differ in their availability norms (Derks et al., 2015; Kreiner et al., 2009). In this study’s context, many employees worked more hybrid hours due to post-COVID-19 restrictions, blurring the physical and mental boundaries between work and private life and making domain integration more likely than before the pandemic.

### 2.2. The Experience of Feeling Interrupted by Personal Smartphone Use During Worktime

Personal smartphone use at work reflects boundary-crossing behavior, potentially becoming habitual and leading to a more fragmented workflow. This study focuses on employees’ subjective experience of being interrupted by such use. Leroy et al. (2020) updated the classical framework by Jett and George (2003) and came up with a more integrated and comprehensive view of interruptions. In their view, interruptions are intrusive when they divert attention from a task despite one’s intention to complete it (Leroy et al., 2020). This can hinder goal progress and cause frustration (Leroy & Glomb, 2018). Notifications indicating personal smartphone messages are hard to ignore due to social and cultural norms to always attend to one’s home roles. These interruptions can be seen as intrusions (explicit demands) or distractions (non-urgent), but both divert attention due to external triggers.

From a different theoretical framework, A. Chen and Karahanna (2014) define technology-mediated interruptions as those occurring via devices (e.g., smartphones or tablets) or apps (e.g., email or texting) without distinguishing between self- or other-initiated sources. In line with Sonnentag et al. (2018) and Derks et al. (2021), we adopt this broad perspective by focusing on employees’ subjective and psychological experience of being distracted or disturbed by personal smartphone messages during worktime without explicitly specifying the cause or type of interruption.

### 2.3. Daily Smartphone Interruptions, Task Accomplishment, and Frustration

Action regulation theory states that interruptions are stressors that have certain costs (Hacker, 2005). Interruptions hinder workflow, thereby undermining goal attainment (Hacker, 2005) and work performance (Bailey & Konstan, 2006). Baethge and Rigotti (2013) argue that on days with too many interruptions, task accomplishment comes at a risk, and some tasks remain unfinished. Or, in occupations where tasks must be finished on the same day, interruptions may result in reduced performance quality. This might be associated with feelings of powerlessness, frustration, or dissatisfaction due to reduced performance quality and quantity (Baethge & Rigotti, 2010, 2013).

Additionally, coping with interruptions during the workday requires effort, which interferes with job performance (Puranik et al., 2020). This relation is even stronger when interruptions originate from a nonwork-related domain (Baethge et al., 2015). As Puranik et al. (2020) show in their integrative review of working interruptions, multiple pathways can explain why and how interruptions interfere with task performance. Our current focus is on the affective pathway: because interruptions hinder goal progress and reduce task completion during the day, they may have adverse affective implications in terms of frustration (Puranik et al., 2020).

To further elaborate on this affective pathway, we build on the Affective Events Theory (Weiss & Cropanzano, 1996) to explain how daily smartphone interruptions at work can hinder goal-directed behavior, undermining task accomplishment—a key source of progress and competence—and thereby trigger negative emotional responses such as frustration. There is some initial empirical evidence that supports this link: interruptions have been associated with irritation and stress (Konradt et al., 2003; Mark et al., 2008; Baethge & Rigotti, 2013), and technology-mediated interruptions have been linked to negative effects via increased time pressure (Sonnentag et al., 2018). Interruptions disrupt the workflow and raise stress levels (Marulanda-Carter & Jackson, 2012), making daily performance goals harder to achieve and provoking adverse affective reactions like anger and frustration (Eatough et al., 2016; Gabriel et al., 2011; Lazarus, 1991). Intrusions not only impede task progress but also disrupt the positive state of flow (P. Y. Chen & Spector, 1992), with frustrated goal attainment being a central mechanism (Leroy & Glomb, 2018). Recent experimental work further shows that reducing interruptions improves performance and reduces irritation (Ohly & Bastin, 2023). Taking this evidence together, we propose that daily smartphone interruptions relate to end-of-day frustration due to their interference with task accomplishment.

**Hypothesis** **1.** *Daily smartphone interruptions are indirectly related to daily frustration via reduced task accomplishment.*

### 2.4. Frustration as an Explanatory Mechanism in the Relationship Between Task Accomplishment and Work-Related Rumination

Work-related rumination refers to “a thought or thoughts directed to issues relating to work that is/are repetitive in nature” (Cropley & Zijlstra, 2011, p. 6). Taking this definition as a starting point, Cropley and Zijlstra (2011) broke with the tradition of recovery scholars (e.g., Sonnentag & Fritz, 2007) in that thinking of work or being unable to switch off from work after work hours is per definition harmful for employees. Within this line of reasoning, Cropley and Zijlstra (2011) conceptualized work-related rumination as a three-dimensional construct: affective rumination (repetitive negative thoughts about work), problem-solving pondering (reflective thoughts about work-related challenges, which might be fulfilling), and psychological detachment (not thinking about work during off-job hours; Sonnentag & Bayer, 2005).

While psychological detachment has received considerable empirical attention, studies that incorporate all three dimensions remain scarce (Weigelt et al., 2019a). To address this gap, our study includes all three forms of work-related rumination. Kinnunen et al. (2017) are one of the few exceptions, showing that work-related interruptions at home negatively impacted psychological detachment but no other forms of rumination. Although their context is similar, we focus on personal smartphone use during work hours as a form of boundary-crossing, studying its relationship with frustration and work-related rumination from a micro-perspective, capturing day-level dynamics.

Employees may continue thinking about work after hours for various reasons, including job stressors (Sonnentag, 2011), unfinished tasks (Syrek et al., 2017), unfulfilled work goals (Smit, 2016), or negative work events (Bono et al., 2013). According to Weigelt et al. (2019b), unfinished tasks evoke a sense of failure and have a negative effect, contributing to work-related rumination. We extend this by examining whether frustration at the end of the workday mediates the relationship between reduced task accomplishment and all three types of work-related rumination in the evening.

Drawing on the Affective Events Theory (Weiss & Cropanzano, 1996), we propose that low task accomplishment triggers frustration—an affective signal of hindered goal progress (Carver & Scheier, 1990)—which then spills over into the evening. This emotional residue may impair psychological detachment and trigger both affective rumination and problem-solving pondering. Prior research supports this: problem-solving pondering often follows unresolved work issues (Cropley & Zijlstra, 2011), whereas successful task completion reduces the need for further mental processing and problem-solving pondering (Sonnentag & Bayer, 2005). In summary, we argue that reduced task accomplishment during the workday increases evening rumination and hinders detachment through heightened frustration.

**Hypothesis** **2.** *The negative relationship of task accomplishment with affective rumination (2a) and problem-solving pondering (2b) and its positive relationship with psychological detachment (2c) in the evening is mediated by frustration at the end of the workday.*

## 3. Method

### 3.1. Procedure and Participants

For this quantitative diary study with repeated daily measures, we recruited a sample of working individuals who owned a smartphone, which they use for private purposes. Participants were primarily recruited via WhatsApp through the researchers’ personal networks. These initial contacts were then asked to disseminate the study invitation within their own networks, resulting in a snowball sampling process and a heterogeneous sample. The study was presented as an investigation into personal smartphone use during work hours, requiring participants to complete brief questionnaires over five consecutive workdays.

Inclusion criteria were as follows: (1) current employment, (2) ownership of a smartphone used for private purposes (a separate device was not required), and (3) working at least four days per week—preferably five—to maximize person-day data. Eligible participants who consented received an email with an informed consent form outlining the study procedure, data confidentiality, and their right to withdraw at any time without explanation.

To track responses longitudinally, participants created a personal code for each questionnaire. This code was used solely for data matching and was removed prior to analysis. Data collection was conducted via Qualtrics, a smartphone-friendly online survey platform. Given the study’s focus on workdays—and considering the prevalence of part-time work in the Netherlands—each daily questionnaire began by asking whether the participant had worked that day; if not, the survey ended immediately. The first questionnaire also collected demographic and background data.

In consultation with each participant, a representative workweek was selected for participation. During that week, participants received daily survey links each evening around 8 p.m., with instructions to complete the questionnaire as late as possible, ideally just before bedtime.

Overall, 91 employees participated in the study. However, since we were interested in daily fluctuations, we excluded participants who participated only once. This resulted in a final sample of 86 participants. Approximately 91% of the participants participated for 4 or 5 days, resulting in 354–399 observations at the within-person level. Participants were predominantly female (58%), and the mean age was 31.6 years (*SD* = 13.09). The sample was mainly highly educated: 69.7% had completed applied sciences successfully or received a university degree. The sample was very diverse in terms of the industries participants worked in. The largest representation came from management (10.5%), architecture and engineering (10.5%), office support and administration (9.3%), life, physical, and social sciences (9.3%), and computers and mathematics (7%).

### 3.2. Measurement Instruments

Demographics were measured in the first daily survey and consisted of gender, age, educational level, and job title. All other variables included in the study were measured on the day level. Items were, if necessary, adjusted to the day level by making a reference to today in the items. Additionally, in the introduction to the items, we made it very clear that the items applied to the current workday or, in the case of rumination, the current evening.

Interruptions by personal smartphone use during worktime were measured using the 3-item scale reported by Derks et al. (2021). An example item is the following: “Today I was often distracted by personal messages on my smartphone during work”. All items were rated on a 5-point Likert scale with the anchors ranging from “totally disagree” (1) to “totally agree” (5). Cronbach’s alpha ranged from 0.82 to 0.93 over the days, with an average of 0.88.

Task accomplishment was measured using the 4-item scale of Ohly and Schmitt (2015). An example item is as follows: “Today, I completed my tasks successfully”. Items were rated on a 5-point Likert scale ranging from “strongly disagree” (1) to “strongly agree” (5). Cronbach’s alpha ranged from 0.81 to 0.92 over the days, with an average of 0.86.

Frustration was measured with the 3-item scale of Peters et al. (1980). An example item is the following: “Today, trying to get my job done was a very frustrating experience”. Items were rated on a five-point Likert scale ranging from “strongly disagree” (1) to “strongly agree” (5). Cronbach’s alpha ranged from 0.51 to 0.72, with an average of 0.62 over the days.

Work-related rumination was measured using the 15-item scale of Cropley et al. (2012). This scale consists of 3 subscales representing the three types of rumination, namely, affective rumination, problem-solving pondering, and psychological detachment. In the introduction for respondents, we emphasized that the items should be applied based on the participant’s free time after work (i.e., the evening). All items were rated on a 5-point Likert scale with the anchors ranging from “totally disagree” (1) to “totally agree” (5). Example items are as follows: “Today I became tense when I thought about work-related issues during my free time” (affective rumination), “This evening, in my free time, I found solutions to work-related problems (problem-solving pondering), and “This evening I felt unable to switch off from work” (psychological detachment). Cronbach’s alpha varied, respectively, from 0.87 to 0.95 with an average of 0.91 for affective rumination, from 0.68 to 0.85 with an average of 0.76 for problem-solving pondering, and from 0.81 to 0.90 with an average of 0.85 for psychological detachment.

### 3.3. Strategy of Analysis

This study employed a five-day diary design, resulting in nested data with daily observations (within-person) clustered within individuals (between-person). We included 86 participants, yielding 354 to 399 daily observations. To account for this multi-level structure, we conducted multi-level analyses using MLwiN (Rashbash et al., 2000). Day-level predictors were group-mean-centered to focus on deviations from individual means. To reduce the risk of false positives, control variables were included only when both theoretically justified and statistically associated with the outcome variable of that specific analysis.

## 4. Results

### 4.1. Descriptives Statistics

Table 1 shows the means, standard deviations, and correlations between the demographic and study variables. For all the variables in our model, we calculated the intra-class correlations (ICC1) to examine how the variance was distributed over the within-person and between-person levels of analysis. The results showed that 56% of the variance in smartphone interruptions, 66% of the variance in frustration, 69% of the variance in task accomplishment, 60% of the variance in affective rumination, 60% of the variance in problem-solving pondering, and 74% of the variance in psychological detachment was due to fluctuations on the day level.

### 4.2. Hypotheses Testing

In the first hypothesis, we proposed that interruptions by personal smartphone use are indirectly related to feelings of frustration via reduced task accomplishment. To test this, we conducted a multi-level analysis to find out whether both predictors (smartphone interruptions) and outcomes (frustration) were related to the mediator variable (task accomplishment). However, since we proposed mediation, we first checked whether there is a direct relationship between smartphone interruptions and frustration. The multi-level model containing age as a control variable and smartphone interruptions as the predictor showed no relationship between interruptions and frustration (γ = 0.10, *SE* = 0.08, *t* = 1.32, *p* = ns).

Second, we tested whether interruptions were negatively related to task accomplishment. The results showed that smartphone interruptions were indeed negatively related to task accomplishment (γ = −0.11, *SE* = 0.05, *t* = 2.32, *p* < 0.05). In addition, the predictor model was a significantly better fit to the data than the model only containing the control variable age (∆−2x log = 5.34, *df* = 1, *p* < 0.05).

As a final step, we tested whether task accomplishment was negatively related to frustration. Multi-level analyses, including age as a control variable and task accomplishment as the predictor, showed a negative relationship between task accomplishment and feelings of frustration (γ = −0.52, *SE* = 0.09, *t* = 5.68, *p* < 0.001). Additionally, the predictor model was a significantly better fit to the data than the model only containing the control variable (∆−2x log = 30.56, *df* = 1, *p* < 0.001). Together, these findings indicate there was no direct relationship between interruption and frustration. The Sobel test showed that interruptions were indirectly and positively related to frustration via reduced task accomplishment (*z* = 2.06, *p* < 0.05), finding partial support for Hypothesis 1.

Hypothesis 2 stated that the negative relationship between task accomplishment, affective rumination (2a), and problem-solving pondering (2b), and the positive relationship between task accomplishment and psychological detachment (2c) is mediated by feelings of frustration at the end of the workday. First, we tested the direct relation between task accomplishment and the three types of rumination (see Table 2 for an overview of the results). Multi-level analyses showed that task accomplishment was negatively related to affective rumination (γ = −0.24, *SE* = 0.07, *t* = 3.32, *p* < 0.01; improved fit compared to the model only containing age as a control variable ∆−2x log = 10.87, *df* = 1, *p* < 0.001), unrelated to problem-solving pondering (γ = −0.05, *SE* = 0.07, *t* = 0.69, *p* = ns), and positively related to psychological detachment in the evening (γ = 0.12, *SE* = 0.06, *t* = 2.14, *p* < 0.05; improved fit compared to the null model ∆−2x log = 4.49, *df* = 1, *p* < 0.05).

Having established that task accomplishment related negatively to frustration (see Hypothesis 1), we then examined whether both predictor (task accomplishment) and outcome variables (affective rumination, problem-solving pondering, and psychological detachment) were related to the mediator (frustration). Regarding the relationship between frustration and the three types of rumination (see Table 3 for an overview of the results), we first compared a model containing only the control variable of age with the model containing both age and frustration as predictors of affective rumination. The results showed that frustration at the end of the workday was indeed significantly and positively related to affective rumination in the evening (γ = 0.29, *SE* = 0.04, *t* = 6.84 *p* < 0.001), and the predictor model showed an improved fit compared to the model only containing the control variable (∆−2x log = 44.04, *df* = 1, *p* < 0.001). Then, we repeated this procedure with gender as a control variable and problem-solving pondering as the outcome variable. The results showed that frustration at the end of the workday was indeed significantly and positively related to problem-solving pondering in the evening (γ = 0.11, *SE* = 0.04, *t* = 2.7 *p* < 0.01), and the predictor model showed an improved fit compared to the model that contained the control variable only (∆−2x log = 7.13, *df* = 1, *p* < 0.01). Finally, we compared the predictor model with frustration as the predictor and psychological detachment as outcome variable with the null model of psychological detachment. Analyses showed that frustration at the end of the workday was a significant negative predictor of psychological detachment in the evening (γ = −0.15, *SE* = 0.04, *t* = 4.17 *p* < 0.001), and the predictor model was a better fit to the data than the null model (∆−2x log = 16.95, *df* = 1, *p* < 0.001).

Finally, we tested whether the relationship between task accomplishment and rumination became weaker (partial mediation) or insignificant (full mediation) after adding frustration as an additional predictor to the multi-level equation. Task accomplishment and problem-solving pondering showed no significant direct relationship, so the model for problem-solving pondering reflects an indirect effect model in which task accomplishments relate to frustration; this, in turn, relates to problem-solving pondering after work (*z* = −2.48, *p* < 0.05). Next, when looking at affective rumination as the outcome, after adding frustration as a predictor next to age and task accomplishment, the relationship between task accomplishment and affective rumination became insignificant (γ = −0.10, *SE* = 0.07, *t* = 1.43, *p* = ns). The Sobel test showed a significant full mediation (*z* = −4.52, *p* < 0.001). Finally, after including frustration as a predictor next to task accomplishment, the relationship between task accomplishment and psychological detachment became insignificant (γ = 0.05, *SE* = 0.06, *t* = 0.83, *p* = ns; Sobel test statistic indirect relation, *z* = 3.15, *p* < 0.01). To sum up, these findings largely support Hypothesis 2. The relationship between task accomplishment, affective rumination, and psychological detachment was fully mediated by frustration. Additionally, task accomplishment was only indirectly related to problem-solving pondering via frustration.

## 5. Discussion

The topic of the current study is the interruptive character of personal smartphone use during worktime and its relationship to task accomplishment, feelings of frustration, and work-related rumination in the evening. We built on the work conducted on technology-mediated interruptions (e.g., A. Chen & Karahanna, 2014) and the work on cumulative interruptions hindering demands during the workday (Baethge & Rigotti, 2013). In addition, building on the affective and cognitive pathways of interruption research (Puranik et al., 2020), we examined whether frustration at the end of the workday may serve as the spill over mechanism inducing all three types of work-related rumination (see Cropley & Zijlstra, 2011) in the evening.

Instead of the proposed relationship between personal interruptions and frustration, mediated by reduced task accomplishment, we found only an indirect relationship between interruptions by personal smartphone use during the day and frustration via reduced task accomplishment. In other words, in the current study, feelings of frustration were linked to the drop experienced in task accomplishment associated with personal smartphone interruptions rather than reflecting a direct response to the interruptions themselves. As such, when employees are interrupted by private issues on their smartphones during worktime, their task accomplishment is compromised, and, in turn, they feel frustrated at the end of the day.

In line with earlier work (Smit, 2016; Syrek et al., 2017), we found an initial relationship between task accomplishment during the day and affective rumination and psychological detachment in the evening. However, against our expectations, task accomplishment was not directly related to problem-solving pondering in the evening. This contradicts prior research, which suggested that problem-solving pondering often arises when individuals face unresolved work issues or incomplete task accomplishment (Cropley & Zijlstra, 2011). The absence of a direct relationship between task accomplishment and problem-solving rumination may be understood in light of the nature of personal smartphone use during work hours. It is plausible that when employees choose to engage with personal matters via their smartphones at work, they may implicitly accept the resulting decrease in task accomplishment (Duke & Montag, 2017) as a trade-off for attending home or personal demands. Because employees have already made a conscious decision to prioritize personal needs over work demands during worktime, they may not experience any unresolved work issues that typically trigger problem-solving pondering. Instead, any impact on task accomplishment is more likely to be internalized as frustration, which then indirectly influences this type of rumination. This could explain why frustration serves as a mediator while a direct link between task accomplishment and problem-solving pondering is absent. Additionally, our results showed that on days employees experienced more frustration at the end of the workday, they engaged in more affective rumination as well as problem-solving pondering and were less able to psychologically detach from their work. Frustration was the explaining mechanism in the relationship between task accomplishment, affective rumination, psychological detachment (full mediation), and problem-solving pondering (indirect relation). These findings extend the Affective Events Theory (Weiss & Cropanzano, 1996) by highlighting how discrete daily work events—such as interruptions and reduced task accomplishment—trigger affective responses (i.e., frustration), which, in turn, activate cognitive processes in the evening. In doing so, we integrate affective and cognitive pathways from interruptions and work–home spill over research (i.e., Ilies et al., 2007; Sonnentag & Fritz, 2015), showing that frustration serves as a key emotional conduit through which daytime work experiences relate to employee well-being beyond office hours.

### 5.1. Theoretical Contributions and Practical Implications

#### 5.1.1. Extending the Rumination Literature

These findings contribute to the literature in several ways. Extending the work of Syrek et al. (2017) and Weigelt et al. (2019b), we show that (a lack of) task accomplishment is related to work-related rumination in the evening, with frustration serving as the key explanatory mechanism. Frustration not only fuels affective rumination and hampers psychological detachment (Peters et al., 1980) but is also associated with more constructive cognitive processes, such as problem-solving pondering (Cropley & Zijlstra, 2011).

While affective rumination and lack of detachment have been linked to poor recovery, problem-solving pondering is unrelated to recovery but positively associated with creativity (Vahle-Hinz et al., 2017). Similarly, Weigelt et al. (2019a) found that detachment relates to life satisfaction; affective rumination relates to burnout; and problem-solving pondering relates to work engagement. These findings underscore that it is not work-related thoughts per se but their *content* and *nature* that determine their impact.

#### 5.1.2. Affective and Cognitive Pathways in Interruption Research

Our results also align with the Affective Events Theory (Weiss & Cropanzano, 1996), which posits that goal-disrupting events elicit emotional responses. Building on this, we contribute to research on the affective pathway of interruptions (Sonnentag et al., 2018) by focusing on personal smartphone interruptions—a modern and pervasive workplace phenomenon. We extend prior work by showing how such interruptions evoke frustration and, in turn, trigger cognitive spill over into the home domain through rumination.

Finally, our study conceptually replicates the findings by Hunter et al. (2019), who showed that boundary violations at work hinder goal progress, leading to negative effects and increased family–work conflict. In summary, we advance the understanding of both affective and cognitive pathways in interruption research (see Puranik et al., 2020, for an overview) and highlight how the implications of workplace interruptions can extend beyond work, influencing well-being at home.

#### 5.1.3. Managing Personal Smartphone Use to Enhance Workplace Focus

Many employees integrate work and personal life to maintain autonomy and meet home demands. While this flexibility supports meaningful relationships with significant others during worktime, our study—based on Derks et al. (2021)—shows that personal smartphone use at work is associated with certain costs. Therefore, we advise employees to limit interruptions due to personal smartphone use, especially on highly demanding days, by scheduling interruption-free periods (Baethge et al., 2015) to protect focus and reduce frustration. In addition, time management skills, which can be developed through training, may also mitigate the negative effects of interruptions (Ma et al., 2020).

For many employees, checking smartphones is habitual or even addictive (Oulasvirta et al., 2012), often going unnoticed. Excessive use can hinder the ability to enter or maintain workflow (Montag et al., 2016). Awareness of the potential negative implications of smartphone interruptions is a key first step toward more productive behavior at work.

#### 5.1.4. Organizational Strategies for Managing Personal Smartphone Use

Organizations should set clear guidelines on acceptable personal smartphone use during work hours while respecting employees’ need for home-life accessibility. This can include designated phone-free zones for focused work and scheduled break times for personal smartphone use. Managers play a crucial role by modeling appropriate behavior. Implementing focus hours and using smartphone focus modes may boost productivity, leading to greater fulfillment and improved well-being (Kushlev & Dunn, 2015).

### 5.2. Limitations and Future Research Directions

Although our findings contribute relevant insights, it is important to note key limitations and future opportunities. We identify and reflect on three key issues in this respect.

#### 5.2.1. Study Design and Measurement Constraints

First, to minimize participant burden and reduce dropout, we limited the number of within-person variables. This left no room for additional control variables beyond standard demographics. Including workload as a variable would have been valuable since a high workload may exacerbate the negative effects of smartphone interruptions—leading to greater frustration and an increased risk of evening rumination—while also prompting problem-solving pondering.

Second, we measured all variables at the end of the day. This may have introduced retrospective bias—especially in recalling affective states like frustration, that typically show fading intensity over time. This led to mediators and outcomes being assessed at the same time and may have caused inflated effect sizes. To address this issue, future research should consider assessing key variables at multiple points throughout the day (e.g., post-interruption, end-of-workday, and pre-sleep).

Finally, the internal consistency of our daily frustration measure was relatively low, possibly because we used a short three-item measure to keep the daily survey short. Even though low reliability tends to result in more conservative estimates, future research should carefully consider the frustration measure and consider replacing or extending it.

#### 5.2.2. Contextual and Temporal Factors

We ran this study shortly after the COVID-19 restrictions when many employees were still working from home. This context may have induced elevated stress and blurred work–life boundaries, triggering elevated personal smartphone interruptions and related frustration (Allen et al., 2021; Vaziri et al., 2020). As hybrid work remains common in the Netherlands, caution is needed when generalizing these findings. Future research should explore how remote and hybrid work shapes the frequency and impact of cross-role interruptions. It may also be worth examining whether working from home reduces work-related interruptions but increases personal interruptions (Delanoeije et al., 2019; Wöhrmann & Ebner, 2021).

#### 5.2.3. Underlying Motivations

In this study, we did not explore why employees engaged in private-to-work boundary-crossing behavior despite potential costs. Understanding the motives behind personal smartphone use at work is essential (Hu et al., 2021), as employees may feel pressure to stay available for personal matters (Delanoeije & Verbruggen, 2019). Importantly, such behavior may not be device-specific—when smartphones are unavailable, employees may switch to other devices for nonwork tasks, keeping overall personal activity constant (Heitmayer, 2025). Future research should examine the motivations behind personal smartphone use and whether they are inherently linked to the device.

## 6. Conclusions

In summary, our findings demonstrate that personal smartphone interruptions during work hours not only impede task accomplishment but also foster frustration, thereby contributing to increased work-related rumination after the working day. By extending the research of Baethge and Rigotti (2013) and Puranik et al. (2020), this study elucidates both the affective and cognitive pathways through which workplace interruptions negatively impact employee well-being beyond the workplace with spill over into their personal lives. The practical implications are clear: minimizing interruptions—especially on demanding workdays—through strategies such as designated focus periods and the use of smartphone focus profiles can help preserve task accomplishment, reduce frustration, and ultimately support better well-being. However, recent evidence (Heitmayer, 2025) suggests that simply restricting smartphone use may not reduce overall personal activity during work, as individuals tend to shift these activities to other devices, such as computers. Future research should investigate the underlying motivations for personal technology use at work and whether the effects observed are specific to smartphones or reflect broader patterns of boundary-crossing behavior. Recognizing how personal smartphone interruptions can undermine task accomplishment and fuel frustration-driven rumination reminds us that our actions at work not only impact our performance but also shape our well-being long after the workday ends.

## Figures and Tables

**Table 1:** Means, standard deviations, and correlations for all study variables.

	Mean	Std.	1.	2.	3.	4.	5.	6.	7.	8.
1.Gender (0 = male, 1 = female)	0.58	0.49		-	0.03	0.16	0.05	0.04	−0.45 ***	−0.11
2. Age	31.60	13.09	−0.18 **		−0.01	−0.02	0.01	−0.01	0.00	0.00
3. Smartphone interruptions	2.07	1.06	0.03	−0.09		0.10	−0.11 *	0.20 ***	0.13 *	−0.07
4. Frustration	3.03	1.28	0.07	−0.20 **	0.15 **		−0.20 ***	0.30 ***	0.11 **	−0.15 ***
5. Task accomplishment	3.94	0.78	0.03	−0.13 *	−0.14 **	−0.29 **		−0.24 ***	−0.05	0.12 *
6. Affective rumination	2.07	1.01	0.03	−0.13 *	0.24 **	0.51 **	−0.21 **		0.35 ***	−0.32 ***
7. PS pondering	2.77	0.91	−0.25 **	0.07	0.07	0.13 *	−0.07	0.38 **		−0.27 ***
8. Psychological detachment	3.27	0.71	0.09	0.03	−0.05	−0.29 **	0.19 **	−0.46 **	−0.37 **	

*** *p* < 0.001; ** *p* < 0.01; * *p* < 0.05. Note: N = 86 persons and N = 354–399 occasions. Correlations below the diagonal are between daily variables based on average scores across the five days that the study took place; correlations above the diagonal are the within-person correlations using person-centered variables.

**Table 2:** Multi-level results of the relationships between task accomplishment and affective rumination, problem-solving pondering, and psychological detachment.

	Affective Rumination		Problem-Solving Pondering		Psychological Detachment	
	Predictor Model		Predictor Model		Predictor Model	
	Estimate	*SE*	Estimate	*SE*	Estimate	*SE*
Intercept	2.08 ***	0.08	3.03 ***	0.10	3.26 ***	0.05
Age	−0.01	0.01				
Gender			−0.45 **	0.14		
Task accomplishment	−0.24 **	0.07	−0.05	0.07	0.12 *	0.06
Variance level 2 (employee)	0.39 (40%)	0.08	0.28 (37%)	0.06	0.13 (26%)	0.04
Variance level 1 (day)	0.58 (60%)	0.05	0.48 (63%)	0.04	0.37 (74%)	0.03
−2 Log likelihood	922.055		847.893		728.596	

*** *p* < 0.001, ** *p* < 0.01, * *p* < 0.05. Data points = 354 of 399 cases in use (respondents N = 86, days N = 5). Gender: 0 = male, 1 = female.

**Table 3:** Multi-level results of the relationships between frustration and affective rumination, problem- solving pondering, and psychological detachment.

	Affective Rumination		Problem-Solving Pondering		Psychological Detachment	
	Predictor Model		Predictor Model		Predictor Model	
	Estimate	*SE*	Estimate	*SE*	Estimate	*SE*
Intercept	2.08 ***	0.08	3.03 ***	0.10	3.26 ***	0.05
Age	−0.01	0.01				
Gender			−0.45 **	0.14		
Frustration	0.29 ***	0.04	0.11 **	0.04	−0.15 ***	0.04
Variance level 2 (employee)	0.41 (45%)	0.08	0.28 (38%)	0.06	0.14 (29%)	0.04
Variance level 1 (day)	0.51 (55%)	0.04	0.47 (62%)	0.04	0.35 (71%)	0.03
−2 Log likelihood	888.887		841.248		716.145	

*** *p* < 0.001, ** *p* < 0.01, Data points = 354 of 399 cases in use (respondents N = 86, days N = 5). Gender: 0 = male, 1 = female.

## Data Availability

The data are not publicly available since we did not include this in the informed consent.

## References

[B1-behavsci-15-00871] Addas S., Pinsonneault A. (2015). The many faces of information technology interruptions: A taxonomy and preliminary investigation of their performance effects. Information Systems Journal.

[B2-behavsci-15-00871] Allen T. D., Merlo K., Lawrence R. C., Slutsky J., Gray C. E. (2021). Boundary management and work-nonwork balance while working from home. Applied Psychology.

[B3-behavsci-15-00871] Anderson C., Heinisch J. S., Ohly S., David K., Pejovic V. (2019). The impact of private and work-related smartphone usage on interruptibility. Adjunct proceedings of the 2019 ACM international Joint conference on pervasive and ubiquitous computing and proceedings of the 2019 ACM international symposium on wearable computers (UbiComp/ISWC ‘19 Adjunct).

[B4-behavsci-15-00871] Andreassen C. S., Torsheim T., Pallesen S. (2014). Use of online social network sites for personal purposes at work: Does it impair self-reported performance?. Comprehensive Psychology.

[B5-behavsci-15-00871] Ashforth B. E., Kreiner G. E., Fugate M. (2000). All in a day’s work: Boundaries and micro role transitions. Academy of Management Review.

[B6-behavsci-15-00871] Baethge A., Rigotti T. (2010). Arbeitsunterbrechungen und multitasking *(Work Interruptions and Multitasking)*.

[B7-behavsci-15-00871] Baethge A., Rigotti T. (2013). Interruptions to workflow: Their relationship with irritation and satisfaction with performance, and the mediating roles of time pressure and mental demands. Work & Stress.

[B8-behavsci-15-00871] Baethge A., Rigotti T., Roe R. A. (2015). Just more of the same, or different? An integrative theoretical framework for the study of cumulative interruptions at work. European Journal of Work and Organizational Psychology.

[B9-behavsci-15-00871] Bailey B. P., Konstan J. A. (2006). On the need for attention-aware systems: Measuring effects of interruption on task performance, error rate, and affective state. Computers in Human Behavior.

[B10-behavsci-15-00871] Biddle B. J. (1986). Recent developments in role-theory. Annual Review of Sociology.

[B11-behavsci-15-00871] Bono J. E., Glomb T. M., Shen W., Kim E., Koch A. J. (2013). Building positive resources: Effects of positive events and positive reflection on work stress and health. Academy of Management Journal.

[B12-behavsci-15-00871] Carton A. M., Aiello J. R. (2009). Control and anticipation of social interruptions: Reduced stress and improved task performance. Journal of Applied Psychology.

[B13-behavsci-15-00871] Carver C. S., Scheier M. F. (1990). Origins and functions of positive and negative affect: A control-process view. Psychological Review.

[B14-behavsci-15-00871] Chen A., Karahanna E. (2014). Boundaryless technology: Understanding the effects of technology-mediated interruptions across the boundaries between work and personal life. AIS Transactions on Human-Computer Interaction.

[B15-behavsci-15-00871] Chen P. Y., Spector P. E. (1992). Relationships of work stressors with aggression, withdrawal, theft and substance use: An exploratory study. Journal of Occupational and Organizational Psychology.

[B16-behavsci-15-00871] Cropley M., Michalianou G., Pravettoni G., Millward L. J. (2012). The relation of post-work ruminative thinking with eating behaviour. Stress Health.

[B17-behavsci-15-00871] Cropley M., Zijlstra F. R. H., Langan-Fox J., Cooper C. L. (2011). Work and rumination. Handbook of stress in the occupations.

[B18-behavsci-15-00871] Delanoeije J., Verbruggen M. (2019). The use of work-home practices and work-home conflict: Examining the role of volition and perceived pressure in a multi-method study. Frontiers in Psychology.

[B19-behavsci-15-00871] Delanoeije J., Verbruggen M., Germeys L. (2019). Boundary-role transitions: A day-to-day approach to explain the effects of home-based telework on work-to-home conflict and home-to-work conflict. Human Relations.

[B20-behavsci-15-00871] Deloitte (2017). Global mobile consumer survey 2017, NL edition.

[B21-behavsci-15-00871] Derks D., Bakker A. B., Gorgievski M. (2021). Private smartphone use during worktime: A diary study on the unexplored costs of integrating the work and family domains. Computers in Human Behavior.

[B22-behavsci-15-00871] Derks D., Van Duin D., Tims M., Bakker A. B. (2015). Smartphone use and work-home interference: The moderating role of social norms and employee work engagement. Journal of Occupational and Organizational Psychology.

[B23-behavsci-15-00871] Duke É., Montag C. (2017). Smartphone addiction, daily interruptions and self-reported productivity. Addictive Behaviors Reports.

[B24-behavsci-15-00871] Eatough E. M., Meier L. L., Igic I., Elfering A., Spector P. E., Semmer N. K. (2016). You want me to do what? Two daily diary studies of illegitimate tasks and employee well-being. Journal of Organizational Behavior.

[B25-behavsci-15-00871] Gabriel A. S., Diefendorff J. M., Erickson R. J. (2011). The relations of daily task accomplishment satisfaction with changes in affect: A multilevel study in nurses. Journal of Applied Psychology.

[B26-behavsci-15-00871] Hacker W. (2005). Allgemeine Arbeitspsychologie—Psychische regulation von wissens-, denk- and körperlich arbeit.

[B27-behavsci-15-00871] Heitmayer M. (2025). When the phone’s away, people use their computer to play: Distance to the smartphone reduces device usage but not overall distraction and task fragmentation during work. Frontiers in Computer Science.

[B28-behavsci-15-00871] Hu X. J., Barber L. K., Park Y., Day A. (2021). Defrag and reboot? Consolidating information and communication technology research in IO psychology. Industrial and Organizational Psychology.

[B29-behavsci-15-00871] Hunter E. M., Clark M. A., Carlson D. S. (2019). Violating work-family boundaries: Reactions to interruptions at work and home. Journal of Management.

[B30-behavsci-15-00871] Ilies R., Schwind K. M., Wagner D. T., Johnson M. D., DeRue D. S., Ilgen D. R. (2007). When can employees have a family life? The effects of daily workload and affect on work-family conflict and social behaviors at home. Journal of Applied Psychology.

[B31-behavsci-15-00871] Jett Q. R., George J. M. (2003). Work interrupted: A closer look at the role of interruptions in organizational life. Academy of Management Review.

[B32-behavsci-15-00871] Kinnunen U., Feldt T., de Bloom J., Sianoja M., Korpela K., Geurts S. (2017). Linking boundary crossing from work to nonwork to work-related rumination across time: A variable-and person-oriented approach. Journal of Occupational Health Psychology.

[B33-behavsci-15-00871] Konradt U., Hertel G., Schmook R. (2003). Quality of management by objectives, task-related stressors and non-task related-stressors as predictors of stress and job satisfaction among teleworkers. European Journal of Work and Organizational Psychology.

[B34-behavsci-15-00871] Kossek E. E., Ruderman M. N., Braddy P. W., Hannum K. M. (2012). Work-nonwork boundary management profiles: A person-centered approach. Journal of Vocational Behavior.

[B35-behavsci-15-00871] Kreiner G. E., Hollensbe E. C., Sheep M. L. (2009). Balancing borders and bridges: Negotiating the work-home interface via boundary work tactics. Academy of Management Journal.

[B36-behavsci-15-00871] Kushlev K., Dunn E. W. (2015). Checking email less frequently reduces stress. Computers in Human Behavior.

[B37-behavsci-15-00871] Kühner C., Rudolph C. W., Derks D., Posch M., Zacher H. (2023). Technology-assisted supplemental work: A meta-analysis. Journal of Vocational Behavior.

[B38-behavsci-15-00871] Lazarus R. S. (1991). Emotion and adaptation.

[B39-behavsci-15-00871] Leroy S. (2009). Why is it so hard to do my work? The challenge of attention residu when switching between work tasks. Organizational Behavior and Human Decision Processes.

[B40-behavsci-15-00871] Leroy S., Glomb T. M. (2018). Tasks interrupted: How anticipating time pressure on resumption of an interrupted task causes attention residue and low performance on interrupting tasks and how a “ready-to-resume” plan mitigates the effects. Organization Science.

[B41-behavsci-15-00871] Leroy S., Schmidt A. M., Madjar N. (2020). Interruptions and task transitions: Understanding their characteristics, processes, and consequences. Academy of Management Annals.

[B42-behavsci-15-00871] Lin B. C., Kain J. M., Fritz C. (2013). Don’t interrupt me! An examination of the relationship between intrusions at work and employee strain. International Journal of Stress Management.

[B43-behavsci-15-00871] Ma J., Kerulis A. M., Wang Y., Sachdev A. (2020). Are workflow interruptions a hindrance stressor? The moderating effect of time-management skill. International Journal of Stress Management.

[B44-behavsci-15-00871] Mark G., Gudith D., Klocke U. (2008). The cost of interrupted work: More speed and stress. CHI ‘08: SIGCHI Conference on Human Factors in Computing Systems.

[B45-behavsci-15-00871] Marulanda-Carter L., Jackson T. W. (2012). Effects of e-mail addiction and interruptions on employees. Journal of Systems and Information Technology.

[B46-behavsci-15-00871] Messenger J., Gschwind L. (2016). Three generations of telework: New ICTs and the (R)evolution from home office to virtual office. New Technology, Work and Employment.

[B47-behavsci-15-00871] Montag C., Duke E., Markowetz A. (2016). Toward psychoinformatics: Computer science meets psychology. Computational and Mathematical Methods in Medicine.

[B48-behavsci-15-00871] Nilsen W., Nordberg T., Drange I., Junker N. M., Aksnes S. Y., Cooklin A., Cho E., Habib L. M. A., Hokke S., Olson-Buchanan J. B., Bernstrøm V. H. (2024). Boundary-crossing ICT use–A scoping review of the current literature and a road map for future research. Computers in Human Behavior Reports.

[B49-behavsci-15-00871] Ohly S., Bastin L. (2023). Effects of task interruptions caused by notifications from communication applications on strain and performance. Journal of Occupational Health.

[B50-behavsci-15-00871] Ohly S., Schmitt A. (2015). What makes us enthusiastic, angry, feeling at rest or worried? Development and validation of an affective work events taxonomy using concept mapping methodology. Journal of Business and Psychology.

[B51-behavsci-15-00871] Oulasvirta A., Rattenbury T., Ma L., Raita E. (2012). Habits make smartphone use more pervasive. Personal and Ubiquitous Computing.

[B52-behavsci-15-00871] Patterer A. S., Yanagida T., Kühnel J., Korunka C. (2021). Staying in touch, yet expected to be? A diary study on the relationship between personal smartphone use at work and work–nonwork interaction. Journal of Occupational and Organizational Psychology.

[B53-behavsci-15-00871] Peters L. H., O’Connor E. J., Rudolf C. J. (1980). The behavioral and affective consequences of performance-relevant situational variables. Organizational Behavior and Human Performance.

[B54-behavsci-15-00871] Puranik H., Koopman J., Vough H. C. (2020). Pardon the interruption: An integrative review and future research agenda for research on work interruptions. Journal of Management.

[B55-behavsci-15-00871] Ramarajan L., Reid E. (2013). Shattering the myth of separate worlds: Negotiating nonwork identities at work. Academy of Management Review.

[B56-behavsci-15-00871] Rashbash J., Browne W., Healy M., Cameron B., Charlton C. (2000). MLwiN (version 1.10.006). Interactive software of multi-level analysis.

[B57-behavsci-15-00871] Reyt J., Wiesenfeld B. M. (2015). Seeing the forest for the trees: Exploratory learning, mobile technology, and knowledge workers’ role integration behaviors. Academy of Management Journal.

[B58-behavsci-15-00871] Smit B. W. (2016). Successfully leaving work at work: The self-regulatory underpinnings of psychological detachment. Journal of Occupational and Organizational Psychology.

[B59-behavsci-15-00871] Sonnentag S., Ackerman P. L. (2011). Recovery from fatigue: The role of psychological detachment. Cognitive fatigue: Multidisciplinary perspectives on current research and future applications. decade of behaviour/science conference.

[B60-behavsci-15-00871] Sonnentag S., Bayer U.-V. (2005). Switching off mentally: Predictors and consequences of psychological detachment from work during off-job time. Journal of Occupational Health Psychology.

[B61-behavsci-15-00871] Sonnentag S., Fritz C. (2007). The Recovery Experience Questionnaire: Development and validation of a measure for assessing recuperation and unwinding from work. Journal of Occupational Health Psychology.

[B62-behavsci-15-00871] Sonnentag S., Fritz C. (2015). Recovery from job stress: The stressor-detachment model as an integrative framework. Journal of Organizational Behavior.

[B63-behavsci-15-00871] Sonnentag S., Reinecke L., Mata J., Vorderer P. (2018). Feeling interrupted—Being responsive: How online messages relate to affect at work. Journal of Organizational Behavior.

[B64-behavsci-15-00871] Syrek C. J., Weigelt O., Pfeifer C., Antoni C. H. (2017). Zeigarnik’s sleepless nights: How unfinished tasks at the end of the week impair employee sleep on the weekend through rumination. Journal of Occupational Health Psychology.

[B65-behavsci-15-00871] Vahle-Hinz T., Mauno S., de Bloom J., Kinnunen U. (2017). Rumination for innovation? Analysing the longitudinal effects of work-related rumination on creativity at work and off-job recovery. Work & Stress.

[B66-behavsci-15-00871] Vaziri H., Casper W. J., Wayne J. H., Matthews R. A. (2020). Changes to the work–family interface during the COVID-19 pandemic: Examining predictors and implications using latent transition analysis. Journal of Applied Psychology.

[B67-behavsci-15-00871] Wan M., Shaffer M. A., Lau T., Cheung E. (2019). The knife cuts on both sides: Examining the relationship between cross-domain communication and the work-family interface. Journal of Occupational and Organizational Psychology.

[B68-behavsci-15-00871] Weatherbee T. G. (2010). Counterproductive use of technology at work: Information and communications technologies and cyberdeviancy. Human Resource Management Review.

[B69-behavsci-15-00871] Weigelt O., Gierer P., Syrek C. J. (2019a). My mind is working overtime—Towards an integrative perspective of psychological detachment, work-related rumination, and work reflection. International Journal of Environmental Research and Public Health.

[B70-behavsci-15-00871] Weigelt O., Syrek C. J., Schmitt A., Urbach T. (2019b). Finding peace of mind when there still is so much left undone—A diary study on how job stress, competence need satisfaction, and proactive work behavior contribute to work-related rumination during the weekend. Journal of Occupational Health Psychology.

[B71-behavsci-15-00871] Weiss H. M., Cropanzano R. (1996). Affective events theory. Research in Organizational Behavior.

[B72-behavsci-15-00871] Wendsche J., Lohmann-Haislah A. (2017). A meta-analysis on antecedents and outcomes of detachment from work. Frontiers in Psychology.

[B73-behavsci-15-00871] Wöhrmann A. M., Ebner C. (2021). Understanding the bright side and the dark side of telework: An empirical analysis of working conditions and psychosomatic health complaints. New Technology, Work and Employment.

